# Visual rating of the medial temporal lobe atrophy on magnetic resonance imaging in elderly patients with type I bipolar disorder

**DOI:** 10.1002/alz70856_101683

**Published:** 2025-12-24

**Authors:** Rodolfo Braga Ladeira, Vinicius Ribeiro Leduc, Marcia Radanovic, Fabio L.S. Duran, Homero Vallada, Orestes Vicente Forlenza

**Affiliations:** ^1^ Laboratorio de Neurociências (LIM27), Instituto de Psiquiatria do Hospital das Clínicas da Faculdade de Medicina da Universidade de São Paulo, Sao Paulo, SP, Brazil; ^2^ Universidade Sao Francisco, Bragança Paulista, SP, Brazil; ^3^ Laboratório de Neuroimagem em Psiquiatria (LIM21), Instituto de Psiquiatria do Hospital das Clínicas da Faculdade de Medicina da Universidade de São Paulo, São Paulo, SP, Brazil; ^4^ Programa de Genética e Farmacogenética (PROGENE), Instituto de Psiquiatria do Hospital das Clínicas da Faculdade de Medicina da Universidade de São Paulo, Sao Paulo, SP, Brazil; ^5^ University of Sao Paulo, Sao Paulo, SP, Brazil

## Abstract

**Background:**

Bipolar disorder (BD) is associated with cognitive impairments and structural brain changes, including brain atrophy. This study aims to evaluate medial temporal lobe atrophy (MTA) – through the visual evaluation of hippocampus, choroid fissure, and temporal horn – in elderly individuals with type‐I bipolar BD on magnetic resonance imaging (MRI) and to explore clinical correlates of greater brain involvement.

**Method:**

MTA was visually rated in 57 elderly euthymic individuals with type‐I BD and 24 elderly controls using Schelten's MTA rating scale. Brain images and clinical data were collected from previous studies conducted at the Hospital das Clínicas of the Faculty of Medicine of the University of São Paulo. Three independent researchers experienced in dementia manually evaluated all neuroimages according to the scale.

**Result:**

The BD group showed significantly higher left medial temporal lobe MTA scores (*p* = .024) than controls, indicating more atrophy. When comparing the MTA scores of elderly BD patients using lithium, elderly BD patients not using lithium and healthy controls, the MTA scores for both hemispheres were higher for bipolar patients not taking lithium when compared to controls, with higher left MTA scores for bipolar patients who were not taking lithium than bipolar patients taking this medication.

**Conclusion:**

In our sample of elderly individuals, we have evidence that there are differences between type‐I bipolar disorder and controls regarding the degree of medial temporal lobe atrophy and that it could be attenuated by lithium exposure. Our results are aligned with the results of a previous study evolving voxel‐based morphometry technique within the same sample of patients and thus reinforce the suggestion that lithium may have neuroprotective effects on hippocampal volume and shed some light on the feasibility of the use of semiquantitative visual rating scales on the investigation of structural brain alterations. We intend to expand the evaluation of the sample with the use of other visual scales developed to assess brain atrophy and white matter hyperintensities, which are easy to apply clinically to assess brain atrophy and white matter hyperintensities in MRI images of elderly BD patients and compare them with healthy controls, enabling the mapping of possible affected brain regions.

